# Optimizing the kidney donor pool: transplanting donor kidneys after partial nephrectomy of masses or cysts

**DOI:** 10.3389/fsurg.2024.1391971

**Published:** 2024-04-24

**Authors:** Marina M. Tabbara, Juliano Riella, Javier Gonzalez, Jeffrey J. Gaynor, Giselle Guerra, Angel Alvarez, Gaetano Ciancio

**Affiliations:** ^1^ Department of Surgery, University of Miami Miller School of Medicine, Miami, FL, United States; ^2^ Miami Transplant Institute, University of Miami Miller School of Medicine, Jackson Memorial Hospital, Miami, FL, United States; ^3^ Servicio de Urología, Unidad de Trasplante Renal, Hospital General Universitario Gregorio Marañón, Madrid, Spain; ^4^ Department of Medicine, University of Miami Miller School of Medicine, Miami, FL, United States; ^5^ Department of Urology, University of Miami Miller School of Medicine, Miami, FL, United States

**Keywords:** kidney transplantation, kidney mass, kidney cyst, living donor kidney donation, donor pool expansion, deceased donor kidney donation, partial nephrectomy

## Abstract

**Background:**

A limiting factor in expanding the kidney donor pool is donor kidneys with renal tumors or cysts. Partial nephrectomy (PN) to remove these lesions prior to transplantation may help optimize organ usage without recurrence of malignancy or increased risk of complications.

**Methods:**

We retrospectively analyzed all recipients of a living or deceased donor graft between February 2009 and October 2022 in which a PN was performed prior to transplant due to the presence of one or more concerning growths. Donor and recipient demographics, perioperative data, donor allograft pathology, and recipient outcomes were obtained.

**Results:**

Thirty-six recipients received a graft in which a PN was performed to remove suspicious masses or cysts prior to transplant. Majority of pathologies turned out to be a simple renal cyst (65%), followed by renal cell carcinoma (15%), benign multilocular cystic renal neoplasm (7.5%), angiomyolipoma (5%), benign renal tissue (5%), and papillary adenoma (2.5%). No renal malignancy recurrences were observed during the study period (median follow-up: 67.2 months). Fourteen complications occurred among 11 patients (30.6% overall) during the first 6mo post-transplant. Mean eGFR (± standard error) at 36 months post-transplant was 51.9 ± 4.2 ml/min/1.73 m^2^ (*N* = 23). Three death-censored graft losses and four deaths with a functioning graft and were observed.

**Conclusion:**

PN of renal grafts with suspicious looking masses or cysts is a safe option to optimize organ usage and decrease the kidney non-use rate, with no observed recurrence of malignancy or increased risk of complications.

## Introduction

Kidney transplantation remains the gold standard treatment for patients with end-stage renal disease (ESRD), with improved overall survival when compared to waitlisted patients remaining on dialysis (1). According to the Scientific Registry of Transplant Recipients (SRTR), in 2021 approximately 122,000 patients were on the waiting list for a deceased donor kidney transplant, and only 25,488 kidney transplants were performed that year (19,518 from deceased and 5,970 from living donors) (2). Clearly, a large discrepancy exists between the number of patients waiting for a kidney transplant and the available donor organ pool. Additionally, 16% of kidney allografts that were initially retrieved in 2020 were not used for transplantation. The reason for such a high non-use percentage is multifactorial and can be organ-, donor-, or recipient- related (3). Renal cysts have been reported to be prevalent in up to 27% of the general population (4). It is difficult to discern with the naked eye that suspicious lesions are simply renal cysts, and studies have shown that the presence of a simple renal cyst increases the risk of infection, bleeding, and conversion into malignancy in the long-term (5). Unroofing these cysts adds an increased risk of urinary leak if connected with the collecting system (6, 7). Renal tumors such as renal cell carcinoma (RCC) were identified in 5 of 553 deceased donor kidneys (0.9%) at the time of organ recovery (8). Performing a partial nephrectomy (PN), also known as nephron-sparing surgery, to remove the cyst or concerning mass with safe margins prior to transplant might assist in optimizing organ usage without increasing the risk of malignancy occurrence or surgical complications developing in the kidney transplant recipient.

**Figure 1:**
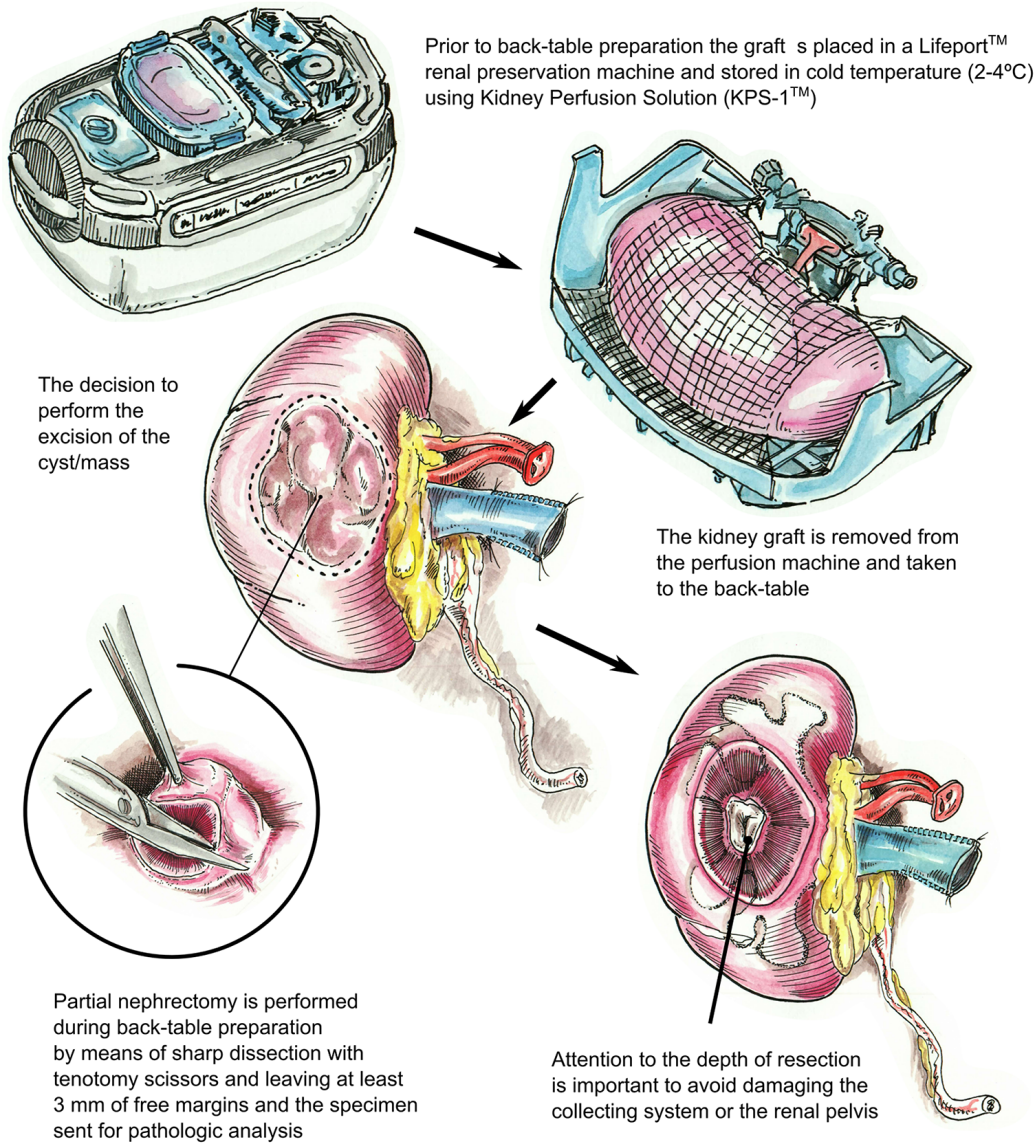
Description of the surgical technique utilized for partial nephrectomy of the deceased donor kidney graft (right kidney). After removing the kidney from the LifePort™ renal preservation machine, if a cyst or mass with suspicious features is identified, a partial nephrectomy is performed during back-table preparation using tenotomy scissors with 3 mm free margins. The specimen is then sent for pathologic analysis. The biopsy site is inspected, as deep dissections can injure the collecting system or the renal pelvis, warranting repair.

**Figure 2:**
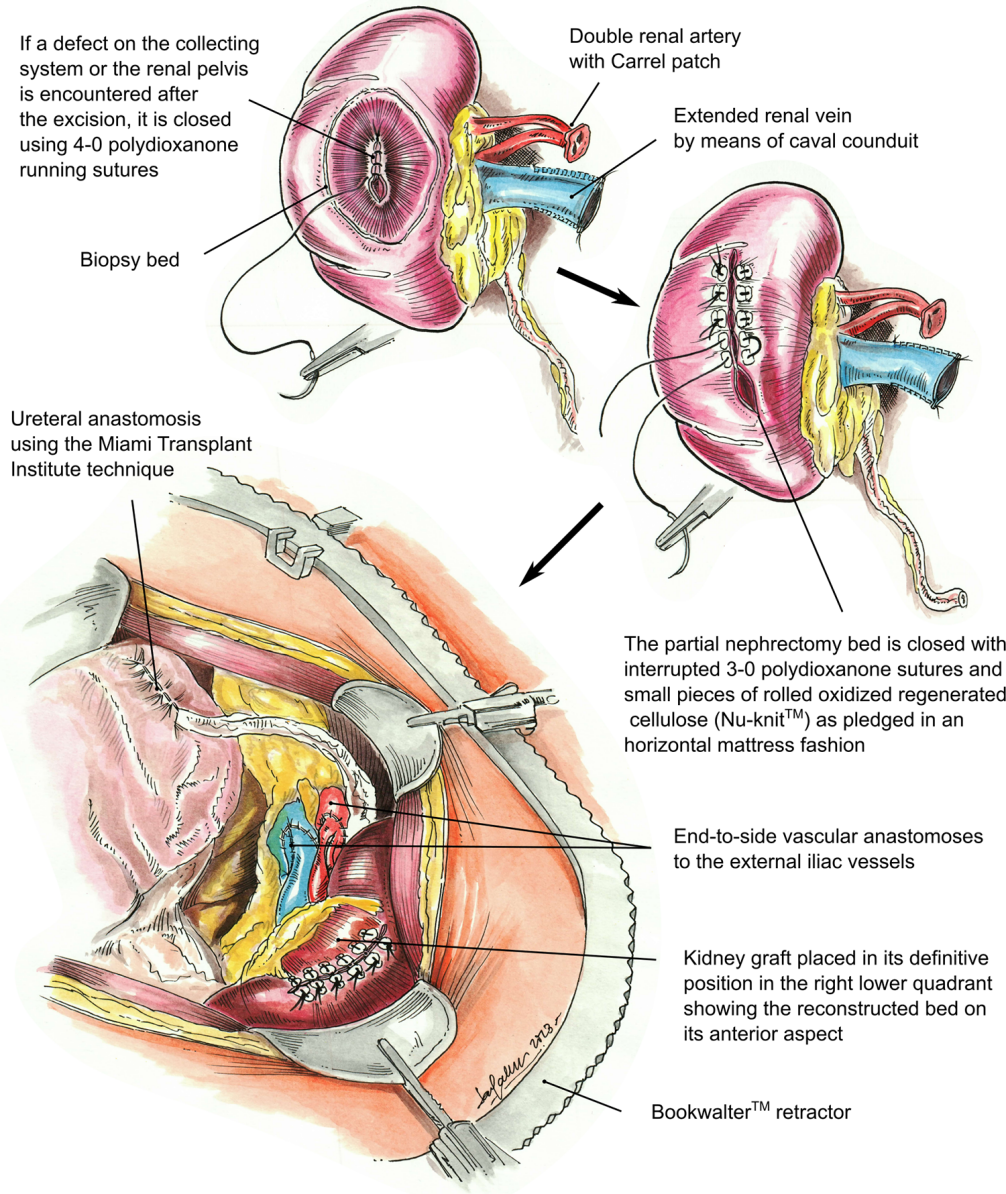
Illustration of the partial nephrectomy site closure of a kidney graft, including repair of the collecting system or renal pelvis defect, followed by retroperitoneal placement of the transplanted kidney in the right lower quadrant. Vascular anastomosis to the right external iliac artery and vein. The renal artery is anastomosed first, followed by the renal vein. Ureteral anastomosis to the recipient bladder using the Miami Transplant Institute extravesical ureteroneocystostomy technique is then performed.

In this study, we analyzed all cases in which a PN was performed to remove one or more suspicious cysts or masses of the kidney allograft prior to transplantation. We describe our surgical technique in detail (Figures 1, 2) and report pathology results, the development of post-operative complications, recurrence rates, and overall kidney transplant recipient outcomes.

## Methods

We retrospectively analyzed all renal transplant recipients of a deceased or living donor graft that underwent a PN between February 2009 and October 2022 due to presence of one or more cysts or masses with a concerning gross appearance. This back table PN was performed in the attempt to prevent: (1) transplantation of a graft with malignancy, (2) the conversion of a complex, possibly benign mass into a malignant one due to the recipient's increased risk secondary to immunosuppression, (3) surgical complications involved with unroofing, decortication, or fulguration, and (4) the recipient or living donor's need for either a radical nephrectomy due to malignancy or active surveillance due to the presence of a complex cyst.

All recipients underwent extensive pre-transplant workup including immunological, medical, and surgical risk assessment. All living donor cases had known renal masses/cysts prior to donation as detected on pre-operative imaging. All living donors were informed of their diagnosis and the possibility of malignancy. If they opted for donation, they would be undergoing the necessary curative treatment for malignancy as the tumor was considered to be small in size. These living donors were offered follow-up with our urology and transplant teams for surveillance post-donation. If the individual opted against donation, they were advised to undergo a biopsy of the mass and were informed, depending on the biopsy results, of the need for possible radical nephrectomy or active surveillance. Deceased donor (DD) kidneys were open or aggressive offers due to the nature of the cyst or mass, and potential recipients of DD kidneys were identified from our DD transplant waiting list based on having no restrictive criteria. Pre-transplant risk of neoplastic transmission was assessed according to the European Best Practice Guidelines and the Kidney Disease Improving Global Outcomes (KDIGO) organization (9, 10). Intermediate-risk organs (defined as 1%–10% risk of neoplastic transmission according to the Disease Transmission Advisory Committee) should be considered for life-saving transplants with patients where the life expectancy without transplant is short (11).

This study was approved by the University of Miami Institutional Review Board and follows the ethical principles (as revised in 2013) of the Helsinki Declaration. Written informed consent was obtained prior to enrollment in this study, which included the potential risks of tumor recurrence, complications related to back-table surgery, and the alternative option of continued dialysis. Specific surgical complications related to excision of the mass (bleeding, urinary leakage, urinary fistula and arteriovenous malformation) were discussed. The United Network for Organ Sharing (UNOS) was also notified of this study and requested a follow-up protocol to be maintained for all patients receiving these allografts. Therefore, we implemented, regardless of malignancy, a doppler ultrasound of the renal graft to be obtained immediately after surgery and repeated every 6 months for 2 years followed by a CT scan of the abdomen and pelvis yearly through 5 years in these recipients of PN allografts.

The standard immunosuppression protocol for these patients consisted of induction with 3 mg/kg of anti-thymocyte globulin IV divided into three doses, two doses of basiliximab 20 mg IV, and Methylprednisolone 500 mg IV for three consecutive days followed by a steroid-free maintenance regimen consisting of tacrolimus and mycophenolic acid (12). If the pathology report revealed a malignancy, then we added an mTOR inhibitor to the maintenance regimen of those recipients who received a PN-performed allograft with the corresponding malignancy having been removed.

Donor and recipient demographics, intraoperative data, pathology results, and recipient outcomes including post-operative complications within six months after transplant were obtained. Complications were classified according to the Clavien-Dindo classification (13). The estimated glomerular filtration rate (eGFR) was calculated using the CKD-EPI formula (14). Delayed graft function (DGF) was defined as the need for dialysis within seven days following kidney transplant. Primary graft nonfunction (PNF) was defined to occur in patients with persistent DGF/dialysis dependency after 3 months post-transplant.

### Surgical technique

**Figure 3:**
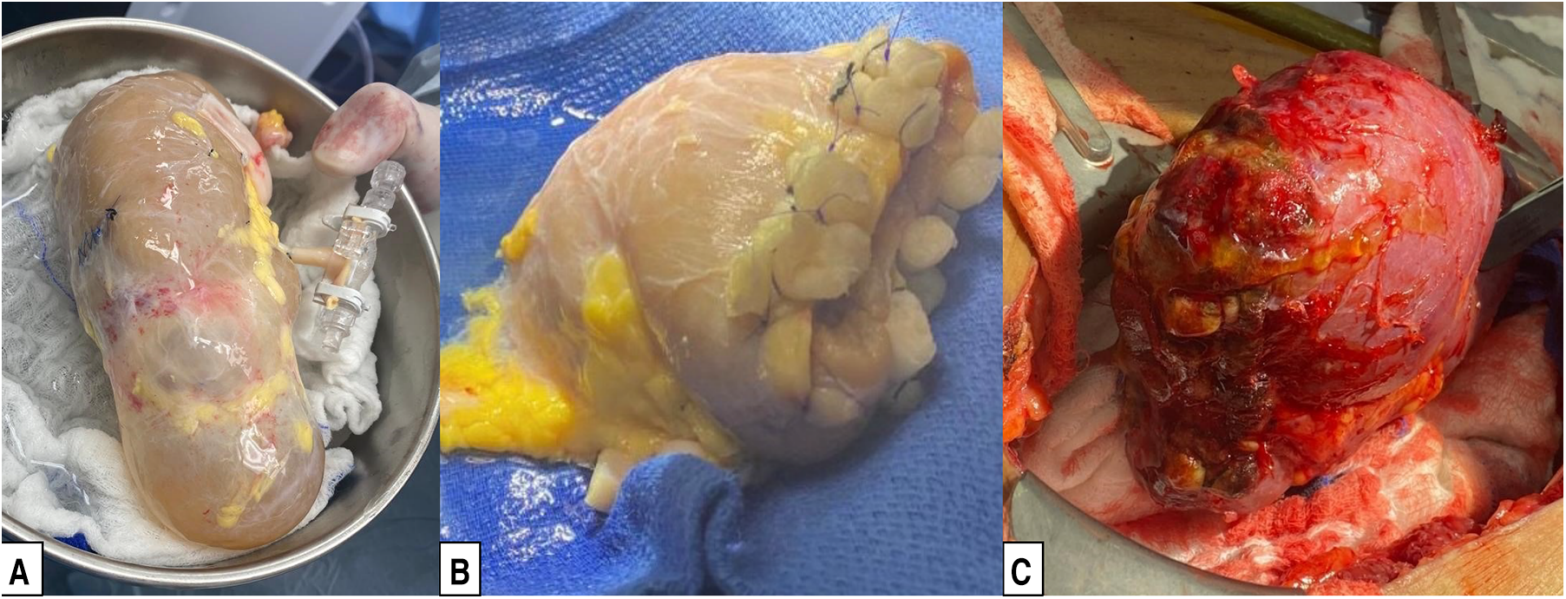
Preparation of a deceased donor kidney with a cyst for transplantation. (**A**) Deceased donor kidney with a large upper pole cyst. (**B**) Deceased donor kidney after partial nephrectomy of the upper pole, as described in the Surgical Technique subsection of the Methods. (**C**) Partial nephrectomy graft after reperfusion.

Prior to back-table preparation, DD kidneys were placed (immediately at the time of arrival at our center) in a Lifeport® renal preservation machine (RPM) and stored in cold temperature (2°C–4°C) utilizing a kidney perfusion solution (KPS-1®) (15) (Figure 1). The decision to perform an excision of the cyst or mass was based on its gross appearance (e.g., complex cyst, solid mass) (Figure 3A). The kidney was completely dissected-free from the peri-renal fat except for the areas in direct contact with the mass or complex cyst. After careful inspection for any additional masses or cysts in the graft, a PN was performed during back table preparation using a sharp dissection with tenotomy scissors, achieving a 3 mm rim of normal kidney parenchyma as margin (Figure 1). Attention to (limiting) the depth of resection is important to avoid damaging the collecting system. If a defect in the collecting system was encountered after the resection, it was closed using 5-0 or 4-0 polydioxanone (PDS®). The kidney cortex was sutured in a continuing manner with 4-0 PDS. The remaining cavity was closed with 4-0 polydioxanone (PDS®) sutures using small pieces of rolled oxidized regenerated cellulose (Nu-knit®) as pledged in a horizontal mattress fashion, and specimens were sent for pathologic permanent analysis (Figures 2, 3B). Before kidney reperfusion, a fibrin sealant (Evicel, OMRIX Biopharmaceuticals Ltd., Israel) is sprayed over the PN site for additional hemostatic properties (Figure 3C). After the removal of the mass or cyst via PN with 3 mm clear margins, margins from the PN were taken for frozen section to ensure that an adequate resection was performed. The entire PN sample(s) was then sent for permanent pathology, and the kidney was immediately transplanted into the recipient. The PN procedure was performed on the back table by an experienced surgeon who utilized judgement that the whole tumor was removed with clear margins, and this was confirmed with the final pathology report.

The kidney transplant surgical technique has been previously described in detail (16). In brief, a modified Gibson incision is performed in the right lower quadrant, and the abdominal wall muscles are divided with an energy sealing device approximately 2 cm from the Linea semilunaris. The peritoneum is reflected medially exposing the iliac vessels. A Bookwalter® retractor is placed to aid in exposure. Minimal dissection is performed of the iliac vessels, and lymphatics are preserved as much as possible. Soft vascular clamps are used for the right iliac external artery, and a Lambert-Kay clamp is used for the iliac vein. The renal artery is anastomosed first using 6-0 Prolene, followed by anastomosis of the renal vein using 5-0 Prolene. Reperfusion of the kidney is then allowed after releasing the vascular clamps with a mean arterial blood pressure of at least 90 mmHg. Then, the ureter is anastomosed to the bladder using the Miami Transplant Institute (MTI) extravesical ureteroneocystostomy technique (Figure 2) (17). After confirming hemostasis, the abdominal wall is closed in two layers, followed by subcutaneous tissue and skin closure. In some cases, a Jackson-Pratt (JP) drain was utilized, but our practice has evolved, and drains have not been routinely used since 2014 (18). Ureteral stents have also been seldomly utilized, as we have demonstrated similar outcomes without the use of ureteral stents if the appropriate surgical technique is used (17, 18).

## Results

Thirty-six patients underwent kidney transplantation with a donor kidney that required a PN. Four kidneys had an additional cyst or mass excised, resulting in a total of 40 pathologies being reviewed. Recipient and donor demographics are described in Table 1. Mean recipient age (± standard error) was 57.6 ± 2.2 years. A majority of recipients were male (72.2%) and of Black (non-Hispanic) race (25.0%) or Hispanic ethnicity (44.4%). Six patients (16.7%) received a pre-emptive transplant; thus, most of the recipients were on dialysis at the time of transplant (83.3%) for a mean dialysis time of 3.57 ± 0.70 years. Mean donor age was 51.4 ± 1.5 years. Thirteen patients (36.1%) underwent living donor transplantation. Twenty-three patients (63.9%) received a graft from a deceased donor. Among 18 deceased donors since 2015, the mean kidney donor profile index (KDPI) was 67.7 ± 4.0% (range: 30%–85%). And among five deceased donors prior to 2015, one was an extended criteria donor. Among all 23 deceased donors, 21 (91.3%) were donation after brain death (DBD). There were 25 (69.4%) grafts with a single renal artery, 10 grafts (27.8%) with two renal arteries, and one graft (2.8%) with three renal arteries. Combining LD and DD recipients, mean cold ischemia time (CIT) was 19.6 ± 2.7 h, and mean warm ischemia time (WIT) was 30.1 ± 0.7 min. Mean percentage of sclerotic glomeruli at the pre-implant biopsy was 6.0 ± 1.0%. A JP drain was used in 20 cases (55.6%), and a ureteral stent was placed in six cases (16.7%).

**Table 1:** Distributions of selected baseline variables (*N* = 36).

Baseline variable	Mean ± SE, along with median and range for continuous variables; percentage with characteristic for categorical variables.
Date of transplant	
2009–2015	47.2% (17/36)
2016–2022	52.8% (19/36)
Recipient age (years)	57.6 ± 2.2 (*N* = 36) [Median = 61.3, Range: 21.0–79.1]
Recipient gender	
Female	27.8% (10/36)
Male	72.2% (26/36)
Recipient race/ethnicity	
Black (non-Hispanic)	25.0% (9/36)
Hispanic	44.4% (16/36)
White (non-Hispanic)	30.6% (11/36)
Preemptive transplant	
No	83.3% (30/36)
Yes	16.7% (6/36)
Time on Dialysis (years)	3.57 ± 0.70 (*N* = 36) [Median = 2.19, Range: 0.00–17.59]
Recipient pretransplant history of diabetes	
No	58.3% (21/36)
Yes	41.7% (15/36)
Donor type	
Living donor	36.1% (13/36)
Deceased donor	63.9% (23/36)
DBD (among 23 DD's)	
No	8.7% (2/23)
Yes	91.3% (21/23)
ECD (among 5 DD's prior to 2015)	
No	80.0% (4/5)
Yes	20.0% (1/5)
KDPI (%)^a^ (among 18 DD's since 2015)	67.7 ± 4.0 (*N* = 16) [Median = 71, Range: 30–85]
Donor age (years)	51.4 ± 1.5 (*N* = 36) [Median = 53, Range: 29–65]
Number of donor arteries	
1	69.4% (25/36)
2	27.8% (10/36)
3	2.8% (1/36)
Cold ischemia time (h)^b^	19.6 ± 2.7 (*N* = 36) [Median = 21.6, Range: 0.2–52.7]
Warm ischemia time (min)	30.1 ± 0.7 (*N* = 36) [Median = 30, Range: 23–40]
Pre-implant Bx: % sclerotic glomeruli	6.0 ± 1.0 (*N* = 29) [Median = 4.0, Range: 0–17]
JP drain use at transplant	
No	44.4% (16/36)
Yes	55.6% (20/36)
Stent inserted at transplant	
No	83.3% (30/36)
Yes	16.7% (6/36)

^a^ KDPI was missing for two simultaenous liver-kidney transplant recipients.

^b^ Cold Ischemia Time includes both living donors (defined by time of transport from donor to recipient on ice) and deceased donors (also defined by time of transport from donor to recipient; however, cold ischemia time included 2 components here: transport from retrieval to placement on machine perfusion, and time on machine perfusion until removal for transplant).

Pathology results and specimen sizes (from the PN) are summarized in Table 2. As mentioned above, 40 distinct pathologic determinations were obtained among the 36 PNs performed among 36 kidney allografts. Of those, 26 (65%) were simple renal cysts. RCC was found in six allografts (15%); of those, three were clear cell type, and three were papillary cell type. Characteristics of the malignancies, including tumor grade, size, and donor type, are summarized in Table 3. Three allografts (7.5%) were found to have benign multilocular cystic renal neoplasm. Two allografts (5.0%) were found to have angiomyolipoma. Two (5.0%) graft biopsies resulted in benign renal tissue without cyst or neoplasm. One (2.5%) graft biopsy was found to have papillary adenoma. Regarding specimen size, 3 (7.5%) measured between 0.1–0.5 cm, 15 (37.5%) measured from 0.6–1.0 cm, 12 (30.0%) measured 1.1–2.0 cm, and 10 (25.0%) measured 2.1–3.0 cm in its largest diameter. Of note, two distinct pathologic determinations were found in 4 of the PNs: a benign multilocular cystic renal neoplasm along a simple renal cyst in one case; an angiomyolipoma along with a simple renal cyst in another case; 2 renal cysts in a third case; and a papillary RCC along with a papillary adenoma in the fourth case.

**Table 2:** Pathology of All suspicious lesions (*N* = 40 pathologies among 36 donor kidneys).

Pathology	Frequency (*n*)	Frequency (%)
Simple renal cyst	26	65
Renal cell carcinoma, clear cell type	3	7.5
Renal cell carcinoma, papillary cell type	3	7.5
Benign multilocular cystic renal neoplasm	3	7.5
Angiomyolipoma	2	5
Benign renal tissue, no cyst or neoplasm	2	5
Papillary adenoma	1	2.5
Total	40	100.0
Specimen size	Frequency (*n*)	Frequency (%)
0.1–0.5 cm in largest diameter	3	7.5
0.6–1.0 cm in largest diameter	15	37.5
1.1–2.0 cm in largest diameter	12	30
2.1–3.0 cm in largest diameter	10	25
Total	40	100

**Table 3:** Characteristics of malignancy cases.

Pathology	Grade	Pathologic size (cm)	Donor type (living or deceased)	Donor age (years)	Recipient age (years)	Clinical follow-up (years)
Renal cell carcinoma, clear cell type	Grade 1	1.2	Living	51	79	11.6
Renal cell carcinoma, clear cell type	Grade 2	0.2	Living	51	55	8.8
Renal cell carcinoma, clear cell type	Grade 1	0.8	Deceased	53	36	5.9
Renal cell carcinoma, papillary cell type	Grade 1	0.6	Living	49	20	4.0
Renal cell carcinoma, papillary cell type	Grade 1	3.2	Deceased	55	69	2.4
Renal cell carcinoma, papillary cell type	Grade 1	0.5	Deceased	47	64	2.3

All specimens were reported with negative margins in the final histologic reports. There was no evidence of any RCC recurrence during the study period. Median follow-up (range) among the 6 patients who received a kidney allograft with RCC in PN pathology specimen was 5.0 (2.3–11.6) years post-transplant (Table 3).

Clinical outcomes are listed in Table 4. We identified six cases (16.7%) of DGF and no cases of PNF. Mean estimated glomerular filtration rate (eGFR) at 3-, 6-, 12- and 36-months post-transplant were 65.0 ± 3.1 (*N* = 36), 61.4 ± 2.7 (*N* = 33), 59.2 ± 3.5 (*N* = 30) and 51.9 ± 4.2 (*N* = 23) ml/min/1.73 m^2^, respectively. Median follow-up among 29 patients with a functioning graft at last follow-up was 67.2 (range 4.3–150.9) months. Three patients (8.3%) developed (death-censored) graft failure - two were related to acute on chronic rejection, and a third patient developed acute rejection due to immunosuppression nonadherence complicated by BK virus nephropathy. A total of four patients (11.1%) died with a functioning graft. The first patient died from COVID-19 pneumonia with respiratory failure at approximately five months post-transplant. The second patient died from a probable cardiovascular event (sudden death) at approximately two years post-transplant. The third patient died of metastatic lung cancer at approximately 5 years post-transplant, and the fourth patient died secondary to trauma from a motor vehicle accident at approximately 6½ years post-transplant.

**Table 4:** Distributions of selected outcome variables (*N* = 36).

Outcome variable	Mean ± SE, along with median and range for continuous outcomes; Percentage developing the outcome for dichotomous outcomes.
Developed DGF	
No	83.3% (30/36)
Yes	16.7% (6/36)
Developed PNF	
No	100.0% (36/36)
Yes	0.0% (0/36)
Developed a 1st post-operative complication^a^	
No	69.4% (25/36)
Yes	30.6% (11/36)
Developed a 2nd post-operative complication^a^	
No	91.7% (33/36)
Yes	8.3% (3/36)
Developed a 1st BPAR^a^	
No	94.4% (34/36)
Yes	5.6% (2/36)
eGFR^b^ at 3 months post-tx (ml/min/1.73 m^2^)	65.0 ± 3.1 (*N* = 36) [Median = 65.5, Range: 34.2–114.5]
eGFR^b^ at 6 months post-tx (ml/min/1.73 m^2^)	61.4 ± 2.7 (*N* = 33) [Median = 55.3, Range: 38.5–92.1]
eGFR^b^ at 12 months post-tx (ml/min/1.73 m^2^)	59.2 ± 3.5 (*N* = 30) [Median = 60.7, Range: 21.2–99.7]
eGFR^b^ at 36 months post-tx (ml/min/1.73 m^2^)	51.9 ± 4.2 (*N* = 23) [Median = 59.2, Range: 12.0–98.3]
Developed (death-censored) graft failure^c^	
No	91.7% (33/36)
Yes	8.3% (3/36)
Death with a functioning graft^c^	
No	88.9% (32/36)
Yes	11.1% (4/36)
Developed (death-uncensored) graft loss^c^	
No	80.6% (29/36)
Yes	19.4% (7/36)
Any death^c^	
No	88.9% (32/36)
Yes	11.1% (4/36)

^a^ Causes (and post-transplant times) of developing a 1st post-operative complication were: Post-operative bleeding (0.07 months), Urine leak from the ureteral anastomosis (0.20 months), Post-operative perirenal collection (0.23 months), UTI (0.36 months), Surgical wound infection (0.36 months), UTI (0.76 months), UTI (0.79 months), Surgical wound infection (1.02 months), Small bowel obstruction (2.04 months), pulmonary aspergillosis (3.42 months), and UTI (5.06 months). Causes (and post-transplant times) of developing a 2nd post-operative complication were: Urine leak from upper pole (0.89 months), Ischemic stroke (1.08 months), and Complicated UTI (2.23 months). Grades (and post-transplant times) of developing a 1st BPAR were: T-cell mediated, Grade 1A (38.4 months), and T-cell mediated, Grade 1B (46.3 months).

^b^ GFR was estimated using the CKD-EPI formula. Also of note, the eGFR for a patient who previously developed kidney graft failure (i.e., returned to permanent dialysis) was not imputed here; thus, patients who previously developed kidney graft failure were not utilized in these mean eGFR calculations.

^c^ As of the last follow-up date, March 1, 2023, death-censored kidney graft failure has occurred in 3 patients [median time to death-censored graft failure was 48.0 (range: 37.5–80.5) months post-transplant]. Causes (and post-transplant times) of death-censored graft failure were: acute and chronic rejection (37.5 months), a combination of BK nephropathy, acute T-cell rejection, and nonadherence (48.0 months), and Acute Rejection (80.5 months). As of the last follow-up date, 4 patients have died with a functioning graft—no patients have died following death-censored graft failure (median time to death with a functioning graft was 43.4 [range: 4.6–77.2] months post-transplant. Causes (and post-transplant times) of death with a functioning graft were: Respiratory failure secondary to COVID-19 pneumonia (4.6 months), Sudden death (probable cardiovascular event, 23.4 months), Lung cancer (63.3 months), and trauma due to a motor vehicle accident (77.2 months). Median follow-up among the 29 patients who were still alive with a functioning graft at last follow-up was 67.2 [range: 4.3–150.9] months post-transplant.

There were 15 complications observed among 11 patients during the first six months after transplant (Tables 4, 5), of which five were surgical-related and two were urologic-related. One patient developed urinary leak from the site of the biopsy of the upper pole of the kidney 26 days after the transplant. This patient was treated with a percutaneous nephroureteral stent placed by interventional radiology, and the urinary leak resolved after 8 weeks. Another patient developed urinary leak from the ureteral anastomosis on postoperative day 6 that was treated with a percutaneous nephroureteral stent, with resolution at 6 weeks post-transplant. This patient then developed a complicated UTI at approximately 2 months post-transplant requiring a prolonged intravenous antibiotic course. One patient developed a perirenal hematoma from a retroperitoneal bleeding vessel requiring exploration on postoperative day 2; the PN site was not the source of the bleeding. This patient subsequently developed an ischemic stroke 30 days after the operation and eventually developed COVID-19 pneumonia with persistent respiratory failure and died at approximately five months after the kidney transplant. Another patient with a previous history of abdominal surgeries developed a high-grade small bowel obstruction two months after transplant and required exploratory laparotomy with lysis of adhesions. Lastly, one patient was diagnosed with pulmonary aspergillosis and was treated with antifungals at approximately 3 months post-transplant.

**Table 5:** Summary of complications observed during the first 6 months post-transplant.

Patient	Drain (Y/N)	Stent (Y/N)	DGF (Y/N)	Complication(s)	Time to occurrence post-transplant (months)	Grade, Clavien-Dindo classification	Treatment
1	Y	Y	N	UTI	0.76	II	IV antibiotics
2	Y	N	N	UTI	0.36	II	IV antibiotics
3	Y	Y	N	Surgical site infection	0.36	IIIb	Debridement in the operating room and IV antibiotics
4	Y	Y	N	UTI	0.79	II	IV antibiotics
5	N	N	N	Small bowel obstruction	2.04	IIIb	Lysis of adhesions in the operating room
6	Y	N	N	Surgical site infection	1.02	II	PO antibiotics
7	Y	N	N	Urinary leak (ureteral anastomosis)	0.20	IIIa	Percutaneous nephroureteral stent by IR
	Y	N	N	Complicated UTI	2.23	II	IV antibiotics
8	Y	N	Y	Perirenal collection	0.23	IIIa	Percutaneous drainage by IR
	Y	N	Y	Urinary leak (upper pole)	0.89	IIIa	Percutaneous nephroureteral stent by IR
9	N	N	Y	Postoperative bleeding	0.07	IIIb	Hematoma evacuation in the operating room
	N	N	Y	Ischemic stroke	1.08	IVa	Medical management
	N	N	Y	COVID-19 pneumonia	1.71	V	Ventilator support in the intensive care unit
10	N	N	Y	UTI	5.06	II	IV antibiotics
11	N	N	Y	Pulmonary aspergillosis	3.42	II	IV antibiotics

## Discussion

Donor organ shortage remains a critical limiting factor for the wider use of renal grafts in transplantation. Several different strategies have been developed in efforts to increase the donor pool, including growth of living donor programs with paired donations and the use of marginal donors, as long as appropriate risks and benefits are considered (19, 20). There is no consensus on the definition of marginal donors, which is also evolving over time; however, marginal donors are usually defined as advanced age donors or those with anatomical anomalies or potentially transmissible infections or malignancies (21). The incidence of donor kidney nonuse in the U.S. is around 24% (22). While the exact numbers of donor kidneys that have been retrieved annually in the U.S. but were not transplanted due to diagnosis of a large cyst or tumor in the donor kidney (or were never retrieved because of their previously detected existence) is unknown, there are only a few centers that have reported on successfully transplanting such donor kidneys when a PN was performed on the back table (3, 23, 24). Our series is relatively large, and in a small way, our approach increased the donor pool over time. Six donor kidneys had pathologies that resulted as RCC, and two donor pathologies resulted as angiomyolipoma. If we had not removed these masses, the donor kidneys simply would not have been used for transplantation. The same goes for those with cysts, as some of them were greater than 2 cm, and such donor kidneys would be discarded or not used (in case of living donors). Thus, performing a PN in these cases allowed us to utilize these particular donor kidneys for transplantation into our recipients.

In the current era of advanced imaging, more incidental renal masses are being diagnosed. These renal masses are often asymptomatic and are found on imaging, ranging from simple renal cysts to RCC (25). RCC is the most frequent malignant renal tumor and one of the most common malignancies worldwide. For small and localized tumors in the general population, nephron-sparing surgery or PN is the indicated surgical approach with good long-term outcomes (26, 27).

The first case of a kidney transplant using a renal graft with excised tumor was reported in 1982 by Stubenbord (28) that was subsequently found to be RCC. Cristea et al. (3) published a review of published case series and case reports that included 147 kidney transplants using grafts that underwent PN, and the pathology was consistent with RCC in 120 of them (81.6%). Only one suspected tumor recurrence was demonstrated. However, that study had several limitations, specifically related to heterogeneity of the obtained data, as most came from different small case series and case reports with only short- to mid-term follow-up. Similarly, Lugo-Baruqui (24) published a review of seven different studies where only one recurrence was encountered in more than 122 recipients transplanted with kidneys that harbored RCC. Desai et al. (29) analyzed more than 17,600 donors, of which 61 of them were determined to have a high risk of cancer transmission. Despite the high risk, they demonstrated a remarkable mean survival of 7.1 years per recipient at 10 years post-transplant, showing that the cancer transmission risk from donors is likely overestimated.

When analyzing solely living donors, Mannami et al. (30) reviewed 42 kidney transplants from living unrelated donors where the graft underwent back table PN of small renal masses. Donor and recipients were followed for up to 135 months post-transplant without RCC recurrence. He et al. (31) also report on their experience with 28 recipients that received a PN kidney graft transplant from living unrelated donors, showing no recurrence of malignancy at a median of 7.5 years of follow up. Regarding living donors with renal cysts, Grotemeyer et al. (32) published a retrospective review of 268 living donor kidney transplants where 25 kidneys were found to have cysts. They reported no cyst-related complications or post-transplant graft dysfunction. In our cohort, 36.1% of the transplants were from living donors, and during our study period, no recurrence of RCC or any other malignancy was encountered.

We report 15 complications that occurred in 11/36 patients during the first 6 months post-transplant. Of these, five were surgery-related, and two additional ones were urological complications. We do not believe that any of non-surgical complications were associated with the PN, as the majority of them were infections that occurred secondary to immunosuppression or comorbidities of the recipient (e.g., the occurrence of ischemic stroke). Regarding surgical complications after PN, Van Poppel et al. (33) published a prospective randomized study comparing PN vs. radical nephrectomy for low-stage RCC and found a rate of urinary fistulas of 4.4% and postoperative bleeding of 3.1% in the PN group. Similarly, a large cohort published in 2004 compared radical nephrectomy and PN (34). Of 361 partial nephrectomies reviewed, the most common complications encountered among the PN recipients were urinary fistulas (5.5%), perinephric abscess (1.1%), acute renal failure (1.3%) and postoperative bleeding (0.8%).

In our cohort, two patients (5.6%) developed a urinary leak. One patient developed a perirenal fluid collection requiring imaged-guided pigtail catheter placement, and after two weeks, a urinary leak was determined to be originating from the upper pole of the transplanted kidney. The second patient developed a leak from the ureteral anastomosis. We do not believe that these urological complications were related to the PN, as one of them was related to the ureterovesical anastomosis, and a second one came from the upper pole, which was at some distance from the PN site (note: this latter complication was likely related to the biopsy site commonly performed and suture-closed by the local Organ Procurement Organization). Both cases were conservatively treated with a nephron-ureteral stent placed by interventional radiology. Our incidence of urological complications (2/36, 5.6%) is similar to previously reported kidney transplants that did not require PN, ranging from 1.7%–15% (35).

Regarding the surgical complications, one small bowel obstruction was encountered, which was most likely due to previous abdominal surgeries and not transplant-related, as the patient's kidney transplant was completely extraperitoneal. There were two surgical site infections, one that required debridement in the operating room, and one that was treated only with antibiotics. These results are similar to the 5% incidence of wound complications occurring among kidney transplant recipients (without a back table PN of the donor kidney) previously reported in the literature (36). Another surgical complication was bleeding requiring hematoma evacuation in the operating room. In this particular case, it came from the retroperitoneum and not from the PN site.

Study limitations include the small sample size, which limits our ability to determine significant risk factors for the development of postoperative complications, and the lack of a comparison group comprised of similar grafts but without the requirement for PN. However, as described above, data from previous studies have shown similar incidences of urologic complications (35, 37) and surgical complications (38, 39), as well as similar clinical outcomes (40).

PN of kidney grafts with suspicious cysts or tumors is a safe approach to optimize organ usage and a good option to expand the donor pool. Our study demonstrates no local or systemic malignancy recurrence with reasonable rates of surgical and urological complications compared to the published literature.

## Data Availability

The raw data supporting the conclusions of this article will be made available by the authors, without undue reservation.
